# A survey of participants in two internet support groups for people with hair-pulling

**DOI:** 10.1186/1471-244X-5-37

**Published:** 2005-10-14

**Authors:** Belinda R Bruwer, Dan J Stein

**Affiliations:** 1 MRC Unit on Anxiety Disorders, University of Stellenbosch, Cape Town, South Africa; 2 Department of Psychiatry, University of Cape Town, Cape Town, South Africa

## Abstract

**Background:**

A substantial number of patients suffering from psychological problems or psychiatric disorders have turned to internet support groups for help. This paper reports on the perceived effectiveness of trichotillomania (TTM) internet support groups for people suffering from hair-pulling.

**Methods:**

A questionnaire was sent via e-mail to all subscribers of two mailing lists devoted to TTM, each of which takes a somewhat different approach to the condition. The questionnaire addressed the possible benefits and problems associated with belonging to a TTM virtual support group.

**Results:**

Subscribers had similar demographic features as clinical samples of trichotillomania patients. Subscribers to both internet lists found them helpful in terms of feeling supported and in obtaining information. The different approaches to TTM on the two lists were associated with differences in treatments attempted by participants.

**Conclusion:**

Internet support groups can potentially contribute to increasing awareness about and knowledge of psychiatric disorders such as TTM, as well as to their management. Nevertheless, additional effort is required to ensure that subscribers are able to make informed, evidence-based decisions.

## Background

A substantial number of people suffering from psychological problems or psychiatric disorders have turned to internet based groups for information and support. Such groups have potential strengths, as well as possible limitations [1]. Previous work has, for example, indicated that virtual groups can be experienced as supportive, but there is also evidence that the quality of medical information on internet websites has limitations [2,3].

In working with people with obsessive-compulsive and spectrum disorders, we regularly encourage patients to seek information from standard websites such as those hosted by the Obsessive-Compulsive Foundation and the Trichotillomania Learning Center. We have previously reported that people with obsessive-compulsive disorder (OCD) can experience virtual OCD groups as both supportive and informative [3].

Although trichotillomania is increasingly recognized to be a prevalent disorder, there are relatively few resources available to those who suffer from this condition [4]. We were interested in the experience that people with trichotillomania (TTM) have of virtual TTM groups. We were intrigued to note that two internet mailing lists (the TTM Telemailer group and the Trichees Yahoogroup) appeared to have somewhat different approaches to this condition, and therefore chose to survey participants in both. The list-owner of the TTM Telemailer has formulated the "John Kender Diet", while the Trichees Yahoogroup seemed to focus on a range of other treatments.

The "John Kender Diet" is based on a hypothesis that people inclined to TTM have a natural biochemistry irreversibly inclined towards certain allergic reactions. The hypothesis is that people with TTM are allergic to yeast (Malessizia sp.) found normally in the skin and gut, and reacts with itching and irritation followed by hair-pulling. Thus, there are "bad" foods and "good" foods and every TTM sufferer should determine his or her optimal diet. "Bad" foods include sugar, egg yolk, peanuts, legumes, butter, shellfish, organ meats, caffeine, nutrasweet, raisins, grapes, watermelon and seeds. "Good" foods include allicins (onions, garlic), tannins, yogurt and tropical fruits [5]. There are no published studies of the efficacy of this diet in trichotillomania.

On the Trichees Yahoogroup listserv, a range of other treatments are discussed, including a "Fish Oil Diet". While essential fatty acids found in fish oil have recently been found effective in controlled trials of a number of different psychiatric disorders [6,7], there are no studies of their efficacy in trichotillomania. In contrast, a number of different medications and psychotherapy interventions have been studied for trichotillomania in randomized controlled trials. Although there are few treatment guidelines for this condition, there is growing evidence for the efficacy of habit reversal, and some patients may benefit from serotonergic medications [4].

## Methods

Two mailing internet lists known to the authors were chosen for the study. The TTM Telemailer was started in March 1995 by John R Kender and had 470 members at the time of the study. The Trichees Yahoogroup was started in October 1998 and had 540 members at the time of the study. Although list-owners do not formally moderate the discussions, they do participate in the discussion, and so are able to influence views of participants.

The project was approved by the Institutional Review Board of the University of Stellenbosch Medical School. A questionnaire was drafted to obtain basic demographic details of subscribers to the mailing lists, and to elicit the perceived benefits and problems of participating in the lists. The questionnaire was informed by a previous survey of participants in an OCD mailing list [3]. With the consent of the list-owners, the questionnaire was sent twice over a three-month period to all list-serve members. Subscribers voluntarily chose whether or not to respond to the questionnaires via e-mail.

A total of 85 questionnaires were received, 58 from the TTM Telemailer (return rate 12.34%), and 27 from the Trichees Yahoogroup (return rate 5%). Four questionnaires were excluded due to incomplete information. Data were tabulated using Microsoft Excel and compared with t-tests or chi-square as appropriate (Table 1&2). At the end of the questionnaire, a section was left for participants to provide any additional comments; the authors read all comments and a representative selection of these is included in the appendix.

**Table 1:** Data from two internet support groups (percentages)

	**Telemailer**	**Trichees**	**chi-square**	**p-values**
**Gender distribution**				
Females	91.2	100	0.985	ns
Males	8.8	0		
**Interest in TTM**				
I have TTM	85.9	87.5	3.647	ns
My child has TTM	8.7	8.3		
Significant other has TTM	1.8	0		
My friend has TTM	1.8	0		
My child and I have TTM	1.8	0		
Significant other and I have TTM	0	4.2		
Mental health professional	0	0		
**Diagnosis and treatment of TTM**				
Professionally diagnosed	61.4	70.8	3.635	ns
Not professionally diagnosed	38.6	29.2		
Treated previously, not presently	38.6	41.7		
Treated previously and presently	22.8	37.5		
Never been professionally treated	38.6	20.8		
**Means of discovering support group**				
Surfing the internet	68.5	91.7	5.402	ns
Support group members	7	0		
Psychologist	3.5	0		
Allied mental health professional	1.8	0		
Friend	1.8	0		
Family member/ significant other	1.8	0		
Additional resources	15.6	8.3		
**Length of time belonging to group**				
More than 12 months	63.1	8.3	29.951	<0.001
6 – 12 months	12.3	20.8		
3 – 6 months	8.8	33.3		
1 – 3 months	12.3	8.3		
1 week to 1 month	3.5	12.5		
Less than 1 week	0	16.7		
**Frequency read and/or respond to messages**				
More than once a day	8.8	8.3	4.676	ns
Once a day	26.3	41.7		
Less than once a day	15.8	20.8		
Weekly	19.3	25		
Monthly	14	0		
**Time spend making use of support group**				
Less than one hour per week	45.6	16.7	6.182	ns
1 – 2 hours per week	31.6	50		
2 – 5 hours per week	15.8	25		
More than 5 hours per week	7	8.3		
**Seeking professional help**				
Support group was very helpful	10.5	15.5	6.548	ns
Only somewhat helpful	26.3	16.7		
Somewhat helpful	8.8	8.3		
Unhelpful	26.3	8.3		
Already sought professional help	28.1	54.2		
**The John Kender Diet**				
Attempted, with good results	33.3	0	18.195	<0.001
Attempted, without good results	19.3	12.5		
Did not attempt the diet	45.6	66.7		
Never heard about the diet	1.8	20.8		
**The Fish Oil Diet**				
Attempted, with good results	0	10	4.600	ns
Attempted, without good results	9.1	40		
Did not attempt the diet	81.8	40		
Never heard about the diet	9.1	10		
**Mislead by information from support group**				
Yes	12.3	4.2	2.480	ns
Maybe	8.8	4.2		
I don't think so	54.4	70.8		
No	24.6	20.8		
**Helpfulness to family and friends**				
Support group was helpful	40.4	33.3	2.004	ns
Support group was unhelpful	19.3	25		
Did not share TTM	31.6	37.5		

**Table 2:** Perceived helpfulness: Data from two internet support groups (percentages)

	**Telemailer**	**Trichees**	**chi-square**	**p -values**
**Learning about symptoms of TTM**				
Helpful	89.5	95.8	0.247	ns
Unhelpful	10.5	4.2		
**Learning about causes of TTM**				
Helpful	77.2	79.2	20.009	ns
Unhelpful	22.8	20.8		
**Learning about treatment of TTM**				
Helpful	82.4	91.6	0.523	ns
Unhelpful	17.6	8.4		
**Tips to decrease hair-pulling**				
Helpful	87.7	79.2	2.977	ns
Unhelpful	8.8	20.8		
Not applicable (I don't have TTM)	3.5	0		
**Feeling supported**				
Helpful	87.7	87.5	2.612	ns
Unhelpful	12.3	8.3		
Not applicable	0	4.2		
**Realizing that you're not alone with TTM**				
Helpful	98.2	91.6	2.849	ns
Unhelpful	1.8	4.2		
Not applicable	0	4.2		
**Giving a name to the hair-pulling problem**				
Helpful	77.2	75	0.409	ns
Unhelpful	21.1	20.8		
Not applicable	1.7	4.2		
**Learning about cognitive- behavioral therapy**				
Helpful	57.9	58.3	0.044	ns
Unhelpful	42.1	41.7		
**Learning about John Kender Diet**				
Helpful	94.8	50	19.534	<0.001
Unhelpful	5.2	50		
**Learning about Fish Oil Diet**				
Helpful	90.9	70	0.439	ns
Unhelpful	9.1	30		

## Results

### Membership and participation

Both groups had similar demographic features: most members were females with hair-pulling who had discovered the support group by surfing the internet, the mean age of subscribers was 32.4 (range 16 – 54), and mean age of onset of TTM was 12.5 (0.5 – 34). A large number of participants (62.5%) were not receiving professional help.

There was a significant difference between the groups in how long people had been members (χ^2 ^= 29.951, p < 0.001); more than 60% of respondents had been members of the TTM Telemailer for longer than 12 months, while more than half of the Trichees Yahoogroup respondents had subscribed within the past 3 to 12 months. Conversely, although most respondents read and or responded to messages posted on the support group once a day, participants in the Trichees Yahoogroup spent significantly more time logged on (Table 1).

### Perceived helpfulness and informativeness

Participants in both lists found these helpful in terms of feeling supported, learning about the symptoms, causes and treatments for TTM, as well as tips on ways to decrease hair-pulling (see Appendix for a range of participant views). The majority of subscribers to both groups believe that they had not been misled by information provided on the support group. More than a third found that the support group had been of help to family and friends. In contrast, a third of participants had not discussed their TTM with family or friends (Table 1).

Participants in both lists found them somewhat helpful in terms of learning about cognitive behavioral therapy and the Fish Oil Diet. The TTM Telemailer was experienced as very helpful in learning about the John Kender Diet, and more participants in this group had tried this diet and found it helpful (Table 1 and 2).

## Discussion

The main findings of this study were that 1) subscribers to both internet lists found them helpful in terms of feeling supported and in obtaining information, and 2) the different approaches to TTM on the two lists were associated with differences in treatments attempted by participants.

Respondents noted in their comments that a great deal of the information provided was useful, as it came from people dealing with the same problem. They also found it useful to interact frequently with someone who had experienced their problem. The internet support groups were helpful in terms of learning about the symptoms and treatment of TTM, tips on how to decrease hair pulling, and in giving a name to their hair-pulling problem. Only a small number of members reported in their comments that their symptoms increased when reading the messages posted on the support group.

Consistent with this positive experience the majority of subscribers were members for more than a year, read and/or responded to messages posted by members once a day or weekly, and spent approximately one to two hours per week making use of the support group. It is notable that many sufferers had not sought professional help, had discontinued treatment, or had not discussed symptoms with family or friends. Indeed, subjects reported having received inadequate information and advice from professionals.

Differences in the apparent content of the two support groups did seem to impact on attempted treatments. The TTM Telemailer subscribers found the support group very helpful in terms of learning about the John Kender Diet, while both support group subscribers found the groups somewhat helpful in learning about the Fish Oil Diet. This may indicate the influence the list-owner has regarding information provided and topics under discussion, as well as attitudes of members towards certain forms of treatments.

Most subscribers had not followed either of these diets or had not achieved good results with them. Nevertheless, people did not feel misled by information, and they continued to participate in the groups. Indeed, members of TTM Telemailer had been subscribers for a longer period than those to Trichees Yahoogroup, suggesting that the former had a longer lasting influence, or alternatively, that people interested in particular nutrition-based interventions were more likely to find this list and to remain subscribers.

Perceived problems related to the support groups included the tediousness of reading and/or responding to large numbers of messages, the abrupt loss of discussion threads when a subscriber suddenly quit the mailing list, the deviation of the discussion away from problems related to TTM, or disagreements with the other subscribers or the list owner's viewpoint. In both groups, English is the language used and this might be difficult for people with English as their second or third language. Another problem mentioned was that members can have different expectations from the support groups, i.e. as a group providing support, as a forum to discuss issues, or as a place to gain information about TTM and recent research.

There are several limitations to the data presented here. First, the sample was a convenience sample; it represents participants who chose not only to continue to participate in the list, but also to answer the questionnaire. Indeed, there was a low response rate, although this might be an underestimation given that not all of the participants in the groups were in fact active members at the time of the study. Second, the survey was not comprised of standardized rating scales, and the extent to which self-report data collected is reliable or valid is unknown. For example, while it seems safe to assume that many participants in the groups had hair pulling, we are unable to confirm that a diagnosis of trichotillomania was in fact present. Possible bias in the survey is compounded by the cross-sectional rather than longitudinal design; there may well be a disjunction between perceived increase or decrease in hair pulling during participation in the survey, and actual changes in symptoms. Participants in the groups have some level of computer literacy, knowledge about how and where to search for the internet support groups, as well as access to a computer and the internet; and the data cannot necessarily be extrapolated to other groups of people with trichotillomania. Nevertheless, membership of the support groups was similar to that described in the clinical literature on hair pulling, i.e. mostly young females [8].

## Conclusion

In conclusion, although there are important limitations to the study, the data here suggest that there may well be a place for internet virtual groups in the management of some people with trichotillomania. Internet lists may also be of value to family, friends and 'significant others' of people with TTM. Mental health professionals may find that participation in internet support groups provides insight into various conditions. Although participants do not feel misled, there is not a focus on evidence-based advice in these TTM lists, and patients should be advised in detail about this issue before being referred to an internet support group. Conversely, subscribers to internet lists should be educated about the potential value of professional evaluation and evidence-based treatment. A systematic evaluation of the effectiveness of internet support groups in reducing hair pulling and distress, and increasing quality of life, is needed.

## Appendix: Comments by subscribers

"This group has helped me to be pull-free for more than 1 month – that is the longest I've ever been pull-free, before this I couldn't even go for one day without pulling."

"The amount of research and knowledge of the participants, as well as practical experiences, has been invaluable in understanding and helping me with trich. The support is amazing!!"

"The only problem is that it's so time consuming to read all the messages and then respond to all the applicable ones..."

"Sometimes I think that talking about it or reading about TTM a lot actually makes me want to pull more..."

"I am involved with someone with TTM... it helps for me to understand what they are all going through and learn from their experiences."

"It is very useful for information on recent research... I live in Australia, and there are very few support groups here, so it's good to be in contact with people all over the world..."

"Over the past seven years, I have found helping the list members very satisfying. I've met electronically, and then sometimes in person, some great people, too."

"The group support has also helped me to confront my feelings about this disorder... and use some of the knowledge I've gained to enlighten family members... and talk about it with friends."

"With partial use of the Diet and a few dealing mechanisms I have been able to stop pulling for 5 months now. I thank this mailer."

"It helps me to understand it better so then it's easier to explain to my friends. Also gave me the confidence to go to a doctor."

"As of today I am 123 days absolutely pull free from scalp hair and 7–8 days pull free from my eyelashes and I have stopped pulling my eyebrows. I might not have come so far if it weren't for this support group."

"The support groups that I belong to have helped me to realize that professional help is VERY limited. The best help you can get is from each other and people like John Kender..."

## Competing interests

The author(s) declare that they have no competing interests.

## Authors' contributions

DS contributed to the concept and design of the study. BB carried out the study, conducted an initial analysis and drafted the manuscript. DS revised the analysis and manuscript. Both authors read and approved the final manuscript.

## Pre-publication history

The pre-publication history for this paper can be accessed here:


